# Hepatic Focal Nodular Hyperplasia: A Benign Incidentaloma or a Marker of Serious Hepatic Disease?

**DOI:** 10.1155/1992/25139

**Published:** 1992

**Authors:** G. Muguti, N. Tait, A. Richardson, J. M. Little

**Affiliations:** 1 Department of Surgery Mpilo Hospital Bulawayo Zimbabwe; 2 Department of Surgery Westmead Hospital Westmead N.S.W. 2145 Australia

## Abstract

Amongst 17 patients with hepatic focal nodular hyperplasia (FNH) encountered at Westmead Hospital between 1981 and 1990, FNH was found in association with hepatocellular carcinoma (HCC) in three (3/ 17), one male and two females, one of whom also had peliosis and an hepatic adenoma. FNH was also found in association with other conditions which may affect hepatic function, structure or circulation, including chronic obstructive airways disease (2), congestive cardiomyopathy (1), chronic active hepatitis (1), granulomatous hepatitis (1), coeliac artery stenosis (1) and metastatic malignant melanoma (1). This report, derived from our experience with FNH over 10 years draws attention to a possible link between FNH, hepatic malignancy and conditions which may disturb the hepatic circulation. We suggest that patients with FNH should be investigated thoroughly and an aggressive management policy should be adopted.


*HPB Surgery*, 1992, Vol. 5, pp. 171–180
Reprints available directly from the publisher
Photocopying permitted by license only

© 1992 Harwood Academic Publishers GmbH
Printed in the United Kingdom

### HEPATIC FOCAL NODULAR HYPERPLASIA: A BENIGN INCIDENTALOMA OR A MARKER OF SERIOUS HEPATIC DISEASE?

G. MUGUTI
*Department of Surgery, Mpilo Hospital, Bulawayo, Zimbabwe*

N. TAIT, A. RICHARDSON and J.M. LITTLE
*Department of Surgery, Westmead Hospital, Westmead, Australia*

*(Received 21 October 1991)*

Amongst 17 patients with hepatic focal nodular hyperplasia (FNH) encountered at Westmead Hospital between 1981 and 1990, FNH was found in association with hepatocellular carcinoma (HCC) in three (3/17), one male and two females, one of whom also had peliosis and an hepatic adenoma. FNH was also found in association with other conditions which may affect hepatic function, structure or circulation, including chronic obstructive airways disease (2), congestive cardiomyopathy (1), chronic active hepatitis (1), granulomatous hepatitis (1), coeliac artery stenosis (1) and metastatic malignant melanoma (1).

This report, derived from our experience with FNH over 10 years draws attention to a possible link between FNH, hepatic malignancy and conditions which may disturb the hepatic circulation. We suggest that patients with FNH should be investigated thoroughly and an aggressive management policy should be adopted.

KEY WORDS: Focal nodular hyperplasia, hepatic neoplasm, liver cancer, liver

### INTRODUCTION

Hepatic focal nodular hyperplasia (FNH) is currently believed to be a benign, usually asymptomatic liver lesion[1]. Its aetiology remains uncertain despite considerable speculation. Factors believed to play an aetiological role include preexisting vascular anomaly, vascular injury (e.g. thrombosis) and long term oral contraceptive medication (OCM) ingestion[2,3,4].

It has been believed that FNH does not undergo malignant change [1]but recently documented associations with liver cancer warrant consideration[3,4,5]. We report a further three cases of hepatocellular carcinoma (HCC) associated with FNH, two of them in long term OCM users.

Address correspondence to: Professor J.M. Little, Department of Surgery, Westmead Hospital, Westmead N.S.W. 2145, Australia

### PATIENTS AND METHODS

The records of all 17 patients admitted to Westmead Hospital with a proven diagnosis of hepatic FNH from January 1981 to January 1990 were reviewed. The following information was extracted from the case records: age, sex, duration of OCM usage, mode of presentation, relevant laboratory results, method of biopsy, method of treatment (conservative or surgical), tumour histology, location and size and long term follow up.

### RESULTS

### *Age and Sex Incidence*

There were 15 females and 2 males with FNH. Median age was 36 years (range 27–67)

### *Clinical Features*

In 6 patients FNH was an incidental finding at operation for some other non-hepatic problem. There were 3 other patients with asymptomatic FNH, 2 picked up during investigation for abnormal liver function tests and one because of an upper abdominal mass found on routine examination. Six patients had symptomatic FNH, causing abdominal pain (4), abdominal discomfort (2), fatigue (1), anorexia (1) or loss of weight (1). Symptoms in the remaining two were due to HCC. There were positive findings on physical examination in 4 patients with FNH (not associated with other pathology); a non-tender upper abdominal mass in 3 and spider naevi and hepatomegaly in one.

### *Oral Contraceptive Medication (OCM)*

Nine of the 15 females had used OCM at some time. Detailed information about OCM usage was available in 7, with a mean duration of OCM usage in these of 10 years (range 4 - 18 years). Two of the 15 females had FNH associated with HCC, one of which was the fibrolamellar variant. Both had been on OCM.

### *Laboratory Results*

None of the patients was anaemic. Liver function tests were available in 16 and in 11 of these there was evidence of impairment of hepatic function. Liver function tests were severely disturbed (raised serum alkaline phosphatase, bilirubin and transaminases) in two patients, both with unresectable hepatic malignancy as well as FNH. In the remaining 9 patients the most common liver function test abnormality was a raised serum alkaline phosphatase. This was elevated to twice the normal level in three patients, one of whom had FNH, HCC, adenoma and peliosis. Another had FNH and chronic active hepatitis and a third had FNH alone. Serum bilirubin was also slightly elevated in the case with FNH and chronic active hepatitis but was normal in all others. In the remaining 6 patients with disturbances of liver function, bilirubin was normal and changes in SAP and transaminases were minor. Serological tests for hepatitis B, alpha-feto-protein and carcino-embryonic antigen were done in 13 patients and were consistently negative.

### *Organ Imaging*

Imaging procedures were not performed in the 6 cases where an unexpected hepatic mass found at laparotomy proved to be FNH. Ultrasound and CAT scanning and angiography were used in the remaining 11. In all, scanning and angiography revealed solid, vascular hepatic lesions, findings consistent with, but not specifically diagnostic of, FNH. Central scars were reported in 4 but found on histopathology in only two of these. On angiography large feeding vessels were noted in 2, spokewheel vascularity in 1. In 2 cases central scarring was found at histopathology which had not been seen on organ imaging. In the 3 cases where FNH and HCC coexisted organ imaging could not differentiate between the two. On IV contrast CAT scanning all 3 show rapid opacification equivalent to that of the adjacent liver parenchyma. Similarly, in one case where FNH and malignant melanoma coexisted, imaging revealed solid, vascular lesions which were not specifically diagnostic and could not be differentiated from each other on the basis of radiological findings.

### *Histopathology*

This was the method of final diagnosis in all but 2 of the 17 cases. A diagnosis of FNH was made on cytology in 2 cases (2/17) early in this series. One of these patients had refused laparotomy, both have had stable lesions on follow-up organ imaging studies. Their FNH studies yielded somewhat dysplastic cells consistent with but not specific for FNH. The remainder underwent laparotomy and open biopsy (15/17) allowing definitive histological diagnosis in 15 of the 17. FNH was associated with HCC in 3 patients; fibrolamellar HCC in one of these, primary HCC in a second and peliosis, liver cell adenoma and primary HCC in the third (Table 1). FNH was seen in the right lobe of the liver in 11 cases, the left lobe in 4 and both lobes in 2. Tumour size was measured by the pathologist in those that were resected and by the radiologist on CT or ultrasound scans in those treated conservatively. Mean tumour size was 7 cm maximum diameter in 12 patients, there being no record of tumour size in five.

**Table 1** Diseases found in association with FNH

| *Intrahepatic* | | *Extrahepatic* | |
|---|---|---|---|
| HCC | 2 | COAD | 3 |
| HCC + Adenoma + Peliosis | 1 | Cardiomyopathy | 1 |
| Melanoma | 1 | Cholelithiasis | 1 |
| Chr. active hepatitis | 1 | Coeliac Artery Stenosis | 1 |
| Granulomatous hepatitis | 1 | Diabetes | 1 |
| | | Gastric Ca. | 1 |

### *Associated Illnesses*

A number of other intrahepatic and extrahepatic conditions which could affect hepatic homeostasis were seen in conjunction with FNH. These are listed in Table 1.

### *Treatment and Follow-up*

Of the 9 patients offered surgical treatment one refused. Eight were offered conservative treatment and are on long term follow up. Tumour size in this group ranged from 1 to 10 cm. Seven of the 8 had single lesions and one had 2 lesions. None of these have shown any change in the size or imaging characteristics of their lesions during follow-up ranging from 1 to 10 years.

One female had inoperable metastatic melanoma diagnosed at laparotomy and her FNH was not resected. One male with inoperable HCC and FNH underwent percutaneous transarterial chemoembolisation using adriamycin and lipiodol. He is on regular follow up with no evidence of progression of either his FNH or HCC 3 months after treatment. One was lost to follow up. Sixteen patients are known to be alive with a median follow up of 3.5 years (range 2 months - 10 years).

### DISCUSSION

The currently accepted view of FNH is of a static, benign liver lesion arising from reparative rather than neoplastic processes[1,6]. This may not be correct. An increasing number of reports associating FNH with primary HCC and its fibrolamellar variant[3,4,5,7] lead to a growing feeling — supported by the material in this paper — that FNH should not be so lightly dismissed. In our series of 17 patients with FNH, six had coexistent hepatic lesions. Three had HCC and one of these also had peliosis and an hepatic adenoma. Two had hepatic inflammatory changes, chronic active hepatitis in one and granulomatous hepatitis in the other. One had hepatic metastases from malignant melanoma.

Five of our patients had extrahepatic illnesses which could disturb hepatic circulation. One had a severe cardiomyopathy with resistent congestive cardiac failure. Another had median arcuate ligament compression with marked stenosis of the coeliac artery. Three had chronic obstructive airways disease, two due to smoking and one due to chronic bronchitis and smoking though none had developed cor pulmonale.

There is a single report of an association between multiple FNH and vascular malformations elsewhere in the body[8]. In addition, patients with multiple FNH in that series developed a higher than expected number of central nervous system tumours[8]. We have not observed this, but we stress that we have not sought it, being unaware of this reported association until recently.

It is difficult to know what the prevalence of FNH is in the community. We know, from pursuing the diagnosis in 64 patients with the chance finding of a mass in the liver on organ imaging that 7 of the 64 had areas of FNH. We have previously reported on 36 of these hepatic incidentalomas[9]. FNH may thus present a diagnostic problem when an unexpected hepatic tumour is found during investigation of other abdominal problems. We stress that there are no clinical, biochemical, serological or organ imaging characteristics nor specific tumour markers which will definitively distinguish FNH from adenoma or HCC[10]. Fine needle aspiration cytology may be helpful, but will commonly produce dysplastic cells compatible with origin in a well differentiated HCC. It is, therefore, imperative that clinicians seek a definitive diagnosis. For these reasons, open biopsy may be necessary.

It is usually taught that FNH should be removed if symptomatic, but otherwise is of no significance[6]. Until now, our management policy has largely followed these traditional guidelines based on a view of FNH as a non-neoplastic, rather inert lesion. However, increasing concern about the association of FNH with malignancy now leads us to suggest that the traditional management regime should be modified. We would now suggest that the diagnosis should be pursued until it is confirmed, and a laparotomy and open biopsy should be carried out. When the diagnosis has been made, a thorough search should be made for malignancy both in the liver and elsewhere in the abdomen. A finding of multiple FNH should initiate screening for vascular malformations elsewhere, and probably cerebral CT scanning to exclude central nervous system tumours[8]. Areas of FNH should be removed wherever possible. Removal should be with a narrow margin of normal tissue, just as if the lesion was a benign neoplasm. If a conservative treatment option is chosen or the lesions are incompletely excised, we now recommend that such patients be regularly screened by organ imaging looking for growth of FNH lesions. Follow-up and screening should be done by a specialist HPB Unit on at least a yearly basis.

The long term history of FNH is not yet known. Our understanding of it is still evolving. It is not clear whether FNH is a marker of some other premalignant change in the liver, or whether it is in itself premalignant. There are suggestions that FNH may progress to fibrolamellar carcinoma,[3] and it is of interest that one of our patients had two areas of FNH adjacent to a fibrolamellar carcinoma. Suspicion that hepatic FNH may be associated with or serve as a marker of more sinister disease is heightened by reports of FNH and HCC coexisting. It is important that clinicians following any number of patients with FNH by regular observation should report their results over many years.

*Acknowledgement*

The authors are grateful to Mrs L. Dahms for typing the manuscript.

*References*

1. Soucy, P., Rasuli, P., Chou, S. and Carpentr, B. (1989) Definitive treatment of focal nodular hyperplasia of the liver by Ethanol embolization. *J. Pediatr.Surg.*, **24**, 1095–1097
2. Ndimbie, O.K., Goodman, Z.D., Chase, R.L., Ma, C.K. and Lee, M.W. (1990) Haemangiomas with localised nodular proliferation of the liver. A suggestion on the pathogenesis of focal nodular hyperplasia. *Am.J.Surg.Pathol.*, **14**, 142–150
3. Vecchio, F.M., Fabiano, A., Ghirlanda, G. *et al.* (1984) Fibrolamellar carcinoma of the liver: The malignant counterpart of focal nodular hyperplasia with oncocytic change. *Am.J.Clin.Pathol.*, **81**, 521–526
4. Saul, S.H., Titelbaum, D.S., Gansler, T.S. *et al.* (1987) The fibrolamellar variant of hepatocellular carcinoma: Its association with focal nodular hyperplasia. *Cancer*, **60**, 3049–3055
5. Serke, S., Dienemann, D., Speck, B., Zimmerman, R. *et al.* (1986) Hepatocellular carcinoma and focal nodular hyperplasia associated with Norethandrolone therapy: A case report. *Blut.*, **52**, 111–116

6. Foster, J.H. (1988) Benign liver tumours, In: Blumgart, L.H. *Surgery of the Liver and Biliary Tract*, 1st Ed. Edinburgh: Churchill Livingstone
7. Berman, M.N., Libbey, N.P. and Foster, J.H. (1980) Hepatocellular carcinoma polygonal cell type with fibrous stroma - An atypical variant with a favourable prognosis. *Cancer*, **46**, 1448–1455
8. Wanless, I.R., Albrecht, S., Bilbao, J. *et al.* (1989) Multiple focal nodular hyperplasia of the liver associated with vascular malformations of various organs and neoplasia of the brain: A new symdrome. *Mod.Pathol.*, **2**, 456–462
9. Little, J.M., Kenny, J. and Hollands, M. (1990) Hepatic incidentaloma: A modern problem. *World J.Surg.*, **14**(4), 448–451
10. Kerlin, P., Davis, G.L., McGill, D.B., Weiland, L.H., Adson, M.A. and Sheedy II, P.F. (1983) Hepatic adenoma and focal nodular hyperplasia: Clinical pathologic and radiologic features. *Gastroenterology*, **84**, 994–1002

*(Accepted by S. Bengmark 22 October 1991)*

### INVITED COMMENTARY

Focal nodular hyperplasia is a relatively rare, apparently benign lesion which occurs in the liver of patients without cirrhosis. It presents as a hepatic mass lesion, single or multiple liver lesion and is becoming increasingly diagnosed. It's occurrence has been related to the use of oral contraceptive but the relationship is less strong than that of hepatic adenoma. Moreover it is found in patients with no history of oral contraceptive use as well as in men. The present series is a ten year review of 17 patients with focal nodular hyperplasia of the liver presenting to a single unit. In the past there has been confusion concerning the nomenclature and histological diagnosis of this condition. Although the features are now well established — a central scarred area surrounded by fibrous bands containing proliferating bile ducts, normal hepatocytes and a degree of chronic lymphocytic infiltration, it might have been useful if the authors had included a brief histological description to define their criteria for the diagnosis.

The existing literature on this condition is littered with case reports, with one or two notable exceptions and so a carefully investigated and followed up series is most welcome. One other series with a 16 year follow up has been reported recently from London[1] but the Australian authors have introduced differing perspectives by first raising the spectre of malignancy associated with focal nodular hyperplasia and second introducing the concept that extrahepatic disease may contribute to its aetiology.

Focal nodular hyperplasia is commonly asymptomatic. More than 50% of the patients studied had the mass lesion discovered incidentally and this is in keeping with other reports. The first problem is reaching a definitive diagnosis. The surgeon may often be called upon to confirm the diagnosis by open biopsy according to the present authors. They were unable preoperatively to confidently discriminate focal nodular hyperplasia from hepatocellular carcinoma in the three cases where the two coexisted, or focal nodular hyperplasia from melanoma metastases using a combination of computerised tomography, ultrasonography and angiography. This was also the experience of Mayo clinic reported 1983[2]. Laparotomy and open biopsy was thus required for diagnosis in 15 of the 17 cases reported. This finding contrasts with more recent studies suggesting that a confident diagnosis can be reached preoperatively in about 80% of cases[1] and in one report without the need for histological confirmation[3]! To rationalise this argument there seems little doubt that the best method of confirming the diagnosis is open biopsy. If the lesion is symptomatic and will need to be removed anyway, then biopsy is unnecessary. If however conservative management or embolisation therapy is being considered then a percutaneous biopsy examined by a pathologist experienced in liver disease should be required.

Inseparable from the diagnostic manoeuvres is the consideration of what should the treatment be? As the authors correctly aver the current view, based on a limited experience of a rare problem, is that focal nodular hyperplasia is rarely symptomatic, will rarely give rise to complications such as bleeding and has a low — if any — potential for malignant transformation. Resection of the lesion or lesions undoubtedly solves the problem and this is attractive given that the majority are in the right lobe and surgery in young patients with normal liver function is attended by a low operative morbidity and mortality. Embolisation has been proposed to avoid the necessity for surgery and on the small numbers reported it seems to be effective[1,3]. The bulk of reported cases have however been managed conservatively and the accumulated data suggest that focal nodular hyperplasia remains static after diagnosis. There are no reported cases of hepatocellular carcinoma occurring in patients undergoing close follow up for this condition. Yet the association of focal nodular hyperplasia with hepatocellular carcinoma is inescapable. It is however something of a "chicken and egg" scenario. The apparent evolution of focal nodular hyperplasia into the fibrolamellar variant of hepatocellular carcinoma has been reported[4]. Indeed the similarities between the two lesions are striking. Almost indistinguishable grossly, they may share a common central scar and fibrous bands[5]. Myelofibroblast like cells can be demonstrated in each on electron microscopy[6] and flow cytometric analyses are very similar[5]. On the other hand hepatocellular carcinoma has not been shown to develop after a diagnosis of focal nodular hyperplasia has been made and there is some evidence to suggest that the latter may occur as a response to alterations in hepatic haemodynamics. For example the kind of alteration which might result from a developing hepatocellular carcinoma. The evidence for altered intrahepatic blood flow causing focal nodular hyperplasia is based around its association with other liver lesions particularly haemangiomas[7]. In autopsy studies the frequency of occult haemangiomas and focal nodular hyperplasia is approximately the same being around 0.3%. It is thought that the hyperplastic nodules develop in response to transient ischaemia. It has also been suggested that the central fibrous scar represents the end stage of a progressive sclerosis/thrombosis of a vascular malformation. The disturbance of haemodynamics associated with intrahepatic lesions is a relatively straightforward concept to grasp. The authors second main point concerning the association of focal nodular hyperplasia with extrahepatic disease is a little more tenuous. Nodular hyperplasia has been associated with a number of systemic disorders which can affect blood vessels, such as rheumatoid arthritis and myeloproliferative disorders. Interestingly there are also associations with immune disorders but no specific reference to acquired immune deficiency. The present series includes one patient with a cardiomyopathy and one with coeliac artery stenosis in whom it is entirely possible that hepatic haemodynamics could be abnormal. In the two patients described with chronic obstructive airways disease, without cor pulmonale, it is less convincing and with such small numbers their speculation on an extrahepatic aetiology remains just that.

In their final recommendations the authors suggest that patients with focal nodular hyperplasia should be investigated thoroughly. This is common sense. Whether or not focal nodular hyperplasia evolves into hepatocellular carcinoma the two are associated and it behoves the clinician to exclude carcinoma before settling for a conservative option.

Focal nodular hyperplasia is becoming more commonly diagnosed and cannot be ignored. Preoperative diagnosis can never be 100% confident. Open biopsy is the best method of confirming the diagnosis and excision biopsy should correct the problem once and for all. The frequency with which focal nodular hyperplasia and hepatocellular carcinoma seem to occur together in resection specimens allows no room for complacency. Embolisation therapy may have something to offer where the diagnosis is unequivocal and there is contraindication to surgery. Otherwise, surgical resection would be the treatment of choice. This will avoid the small but real risk of missing an associated, curable heptaocellular carcinoma.

### *References*

1. Pain, J.A., Gimson, A.E.S., Williams, R. and Howard, E.R. (1991) Focal nodular hyperplasia of the liver: results of treatment and options in management. *Gut*, **32**, 524–527
2. Kerlin, P., Davis, G.L., McGill, D.B., Weiland, L.H., Adson, M.A. and Sheedy, P.F. II (1983) Hepatic adenoma and focal nodular hyperplasia: Clinical, pathologic and radiologic features. *Gastroenterology*, **84**, 994–1002
3. Soucy, P., Rasuli, P., Chou, S. and Carpenter, B. (1989) Definitive treatment of focal nodular hyperplasia of the liver by ethanol embolisation. *J.Paed.Surf.*, **10**, 1095–1097
4. Berman, M.M., Libbey, P. and Foster, J.H. (1980) Hepatocellular carcinoma: Polygonal cell type with fibrous stroma. An atypical variant with a favourable prognosis. *Cancer*, **46**, 1448–1455
5. Saul, S.H., Titelbaum, D.S., Gansler, T.S. *et al.* (1987) The fibrolamellar variant of hepatocellular carcinoma. Its association with focal nodular hyperplasia. *Cancer*, **60**, 3049–3055
6. Vecchio, F.M., Fabiano, A., Ghirlanda, G., Manna, R. and Massi, G. (1984) Fibrolamellar carcinoma of the liver: The malignant counterpart of focal nodular hyperplasia with oncocytic change. *Am.J.Clin.Pathol.*, **81**, 521–526
7. Ndimbie, O.K., Goodman, Z.D., Chase, R.L., Ma, C.K. and Lee, M.W. (1990) Haemangiomas with localised nodular proliferation of the liver. A suggestion on the pathogenesis of focal nodular hyperplasia. *Am.J.Surg.Pathol.*, **14**, 142–150
8. Blumgart, L.H. (ed.) (1988) Surgery of the liver and biliary tract. London: Churchill Livingstone

G.T. Sunderland

A.K.C. Li

Department of Surgery

Prince of Wales Hospital

Shatin, New Territories

Hong Kong

### INVITED COMMENTARY

The malignant potential of the so-called "benign" liver tumors (liver cell adenoma and focal nodular hyperplasia [FNH]) remains in question in spite of 25 years of increasing interest and experience with these rare lesions. The co-incidence of focal nodular hyperplasia with carcinoma in 3 of 17 patients with focal nodular hyperplasia reported by Muguti *et al.* is frightening indeed. Although many surgeons are concerned about the pre-malignant nature of liver cell adenoma, few have recommended resection for FNH to "prevent cancer" once a histologic diagnosis was firmly established. Should this recommendation change?

In spite of considerable interest and an ongoing cooperative effort with others around the world who share that interest, this writer cannot take a strong stand on either side of this question in 1991. My confusion is based upon:

1. Although agreement about histologic differences between liver cell adenoma and FNH are much clearer than they were two decades ago, some tumors have specific features of both lesions, and different pathologists may make different calls.
2. Although nodular lesions with the histologic criteria for malignancy are easy to produce in the livers of experimental animals and have been found in humans undergoing androgen therapy, such lesions may not always have the biologic potential to spread and to kill if untreated.
3. Similarly, the clinical significance of a focus of histologic cancer found within the substance of a resected liver cell adenoma remains unknown. The parallel with thyroid and breast carcinoma is obvious. However, the risk of "prophylactic" organ removal for the liver far outweights that for breast or thyroid.
4. No convincing report of definite malignant change in a solitary benign liver tumor left in place is yet known to this writer. However, at least two patients with proven multiple liver cell adenomas have eventually developed primary liver cancer while under observation, one dying of her disease and the other requiring transplantation.

Perhaps when each and all of the leads suggested by others are brought together, there will be enough evidence to settle this question, but it may turn out to be much like the problem of the colon polyp and cancer.

More specific details about the geography of the benign and malignant components of the tumors in this Australian report would be useful in trying to understand any relationships. Was the cancer within the "benign" tumor or were two discrete lesions separated widely by normal liver tissue?

I had only a few other minor problems with this paper. I do not believe that diagnosis of FNH can be made by aspiration cytology. I know of no evidence that suggests that a margin of normal liver tissue need be taken when resecting a benign liver tumor, although only the hemangioma has a true capsule which allows easy enucleation. And lastly, can one make a diagnosis of focal nodular hyperplasia in a liver diffusely involved with chronic active inflammation and repair? I think not, since nodular hyperplasia is part and parcel of any such process and may be either focal or diffuse.

We should be grateful for the report of this Australian experience. It has raised the importance of resolving this issue. It would be foolish to accept the probability of an association between FNH and primary liver cancer on the basis of this anecdotal evidence. It would be even more foolish to deny the possibility of such an association. Would I recommend liver transplantation for a patient proven to have multiple FNH tumors geographically not amenable to local resection? No, I would not. Not yet.

James H. Foster
Professor
Department of Surgery
University of Connecticut Health Center
Farmington, CR USA 06030

